# Questioning the Yelp Effect: Mixed Methods Analysis of Web-Based Reviews of Urgent Cares

**DOI:** 10.2196/29406

**Published:** 2021-10-08

**Authors:** Dian Hu, Cindy Meng-Hsin Liu, Rana Hamdy, Michael Cziner, Melody Fung, Samuel Dobbs, Laura Rogers, Monique Mitchell Turner, David André Broniatowski

**Affiliations:** 1 Department of Engineering Management and Systems Engineering School of Engineering and Applied Science George Washington University Washington, DC United States; 2 Department of Environmental and Occupational Health Milken Institute School of Public Health George Washington University School of Medicine and Health Sciences Washington, DC United States; 3 Division of Infectious Diseases Children's National Hospital Washington, DC United States; 4 Department of Pediatrics George Washington University Washington, DC United States; 5 Department of Health Policy and Management Milken Institute School of Public Health George Washington University School of Medicine and Health Sciences Washington, DC United States; 6 Department of Communication College of Communication, Arts, and Sciences Michigan State University East Lansing, MI United States

**Keywords:** urgent care, doctor-patient communication, doctor web-based review, review websites

## Abstract

**Background:**

Providers of on-demand care, such as those in urgent care centers, may prescribe antibiotics unnecessarily because they fear receiving negative reviews on web-based platforms from unsatisfied patients—the so-called *Yelp effect*. This effect is hypothesized to be a significant driver of inappropriate antibiotic prescribing, which exacerbates antibiotic resistance.

**Objective:**

In this study, we aimed to determine the frequency with which patients left negative reviews on web-based platforms after they expected to receive antibiotics in an urgent care setting but did not.

**Methods:**

We obtained a list of 8662 urgent care facilities from the Yelp application programming interface. By using this list, we automatically collected 481,825 web-based reviews from Google Maps between January 21 and February 10, 2019. We used machine learning algorithms to summarize the contents of these reviews. Additionally, 200 randomly sampled reviews were analyzed by 4 annotators to verify the types of messages present and whether they were consistent with the Yelp effect.

**Results:**

We collected 481,825 reviews, of which 1696 (95% CI 1240-2152) exhibited the Yelp effect. Negative reviews primarily identified operations issues regarding wait times, rude staff, billing, and communication.

**Conclusions:**

Urgent care patients rarely express expectations for antibiotics in negative web-based reviews. Thus, our findings do not support an association between a lack of antibiotic prescriptions and negative web-based reviews. Rather, patients’ dissatisfaction with urgent care was most strongly linked to operations issues that were not related to the clinical management plan.

## Introduction

The World Health Organization has deemed antibiotic resistance, which is primarily caused by antibiotic overuse, to be one of the world's most pressing health problems [1]. Antibiotic overuse is widespread; approximately one-third of all antibiotics prescribed in US outpatient settings are unnecessary [2]. Care providers have admitted to prescribing antibiotics—even when antibiotics are unnecessary—when they assume that patients will be unsatisfied without an antibiotic prescription [3,4]. However, care providers' assumptions about a patient's expectations frequently do not match patients' actual expectations for an antibiotic [5]. Furthermore, prior literature on patient satisfaction and provider-patient communication have suggested that there are other factors that drive patient satisfaction [6]. For example, Welschen et al [7] found that receiving information or reassurance was more strongly associated with satisfaction than receiving an antibiotic prescription in primary care. Ong et al [8] found that patient satisfaction was not related to the receipt of antibiotics but was related to the belief that patients had a better understanding of their illness. Stearns et al [9] found that patients generally had equal levels of visit satisfaction regardless of their antibiotic treatment status.

Despite findings that do not support a link between patient satisfaction and antibiotics, care providers still report prescribing antibiotics unnecessarily because they fear that dissatisfied patients will leave negative reviews [10]. Authors in several media outlets [11,12] have coined the term *Yelp effect*; they propose that providers of on-demand care, such as those in urgent care centers, may prescribe antibiotics unnecessarily to prevent patients from leaving negative web-based reviews. There is a perception among urgent care providers that many of their patients expect to receive antibiotics, even when they are clinically unnecessary, and that patients will leave negative reviews on web-based platforms if these expectations are not met. To avoid negative web-based reviews, which could impact care providers’ pay and the performance of urgent care centers, care providers are driven to prescribe antibiotics, even when they are clinically unnecessary.

Concerns about the Yelp effect are magnified by the surging popularity of review websites, such as reviews on Google Pages, Yelp, and Healthgrades [11]. Even though most physician ratings on web-based platforms are positive [13,14], at least 1 study has demonstrated that in web-based physician reviews, the words *medication* and *prescription* are mentioned in more negative contexts [15]. This perceived connection between negative web-based reviews and antibiotics is hypothesized to be a significant driver of inappropriate antibiotic prescribing and is evident in at least 1 petition that has received 40,000 signatures supporting the removal of web-based doctor reviews [16].

Concerns about the impact of negative reviews appear to be valid; patients often use web-based reviews when deciding whether and where to seek treatment [17]. Furthermore, these reviews may not accurately reflect the quality of medical care. For example, Daskivich et al [18] conducted an analysis of 5 popular web-based platforms and showed that there was no significant association between web-based consumer review scores and standard quality guidelines. Likewise, Yaraghi et al [19] found that consumers tend to perceive care provider ratings from nonclinical websites to be as important as the ratings from government websites. Thus, web-based reviews can drive care providers’ behaviors in ways that negatively impact public health.

At present, the extent to which patients’ expectations for antibiotic treatment drive negative web-based reviews is unknown. Even though web-based reviews have been mined for other health topics [20,21], to our knowledge, ours is the first study to evaluate web-based reviews and antibiotic prescribing. Thus, we sought to determine whether there truly is a Yelp effect by evaluating how frequently patients leave negative web-based reviews regarding a lack of antibiotic prescriptions.

To determine the prevalence of the Yelp effect, we analyzed a large sample of web-based reviews of urgent care centers in the US by calculating the proportion of negative reviews that exhibit a message regarding a lack of antibiotic prescriptions. Specifically, we sought to (1) quantify the proportion of negative reviews that were posted due to patients (reviewers) not receiving an antibiotic and (2) evaluate the content of negative reviews of urgent care centers.

## Methods

### Data Collection

We used the Yelp application programming interface (API) between October 1 and October 20, 2018, to obtain a list of facilities that were tagged as “urgent care” in the United States. We also retrieved the star ratings for all reviews that were associated with these facilities. We removed all facilities that did not have a US zip code. We were unable to obtain the full texts of these reviews—Yelp's terms of service prohibit web scraping, and Yelp declined to provide us with permission to use the text. Thus, by using the list of facilities that was obtained from the Yelp API, we ran the data collection algorithm from January 21 and February 10, 2019, and obtained all of the Google Maps reviews of each urgent care facility that were posted before January 21, 2019. Unlike Yelp, Google's terms of service permit the collection of their reviews as long as it does not put undue burdens on Google's servers. To collect data from Google Maps reviews, we designed a computer program (Multimedia Appendix 1) for automatically collecting these reviews based on their Google Maps URLs. The Google Maps URLs of the urgent care facilities were collected after we presented their titles and addresses (obtained from the Yelp API) to 20 workers on Amazon Mechanical Turk, a crowdsourcing marketplace. These 20 workers were selected based on their prior demonstrated ability to successfully collect a set of 100 known URLs. Workers received US $0.09 for each URL collected. To summarize the contents of web-based reviews, we used a machine learning algorithm that was designed to summarize text—the latent Dirichlet allocation (LDA) topic model [22]. This algorithm was implemented in the LDA python package [23] (default settings were used) to the fit the model to the Google Maps data set. Topic models identify review topics automatically without human intervention by examining the word co-occurrence statistics within each review [24]. For each topic, we calculated the total number of word tokens that were found in positive reviews (4 or 5 stars) and negative reviews (1 or 2 stars). We fitted 3 topic models (one model with 10 topics, another with 20 topics, and another with 50 topics) to the data. We selected the model that generated the most coherent topics without a large increase in perplexity (a measure of model goodness of fit that is commonly used in natural language processing; Multimedia Appendix 2). Afterward, we extracted antibiotic-related reviews by using a list of keywords that was generated by one of the authors (RH)—a pediatrician who specializes in antibiotic stewardship (see Multimedia Appendix 3 for a keyword list). For each review, we examined the proportion of words in each topic. We applied the same procedure to the subset of reviews containing antibiotic-related keywords. We then developed a qualitative codebook to determine the content and sentiment of 200 reviews that were sampled at random from all reviews containing these keywords. This study was approved by The George Washington University Committee on Human Research Institutional Review Board (Federal Wide Assurance number: FWA00005945; institutional review board registration number: 180804).

### Data Annotation

Four authors (LR, MF, MC, and SD) affiliated with The George Washington University Antibiotic Resistance Action Center collectively reviewed a subset of the 200 randomly sampled reviews to determine the types of messages that were present in the reviews [25]. After this initial review and the development of inductive codes, the reviews were categorized into one of the following categories in the codebook: (1) the *Yelp effect* category (the patient wanted antibiotics but did not receive them); (2) the *opposite of the Yelp effect* category (the patient received antibiotics but did not want them); (3) the *convenience, inconvenience, and wait times* category; (4) the *staff competence or incompetence, courtesy and attitude, and satisfaction of care* category; (5) the *cost and price of drugs per visit (including sticker shock)* category; (6) the *other prescription-related complaints* category; and (7) the *other or none of the above* category. Additionally, all reviews were annotated as “positive” (eg, the patient was satisfied with their care, and the review had 4 or 5 stars) or “negative” (eg, the patient was dissatisfied with care, and the review had fewer than 4 stars).

The four annotators then independently reviewed the same 200 randomly sampled reviews to assign them to 1 of the 7 categories. The final categories for the reviews were assigned based on the majority category among annotators. If there was no majority category, disagreements were resolved discursively until a consensus category was agreed upon.

Some reviews mentioned that the reviewer did not receive an antibiotic when it was expected, even if that was not the main message. Thus, after assigning each message a primary category, the same four annotators revisited all 200 reviews to determine if they mentioned the Yelp effect in passing (ie, whether the review mentioned an unfulfilled expectation for antibiotics). A code for mentioning the presence or absence of the Yelp effect—even if it was mentioned in passing—was then assigned to each review as a secondary code. By using these 200 annotated samples, we inferred population proportions for each category in our codebook and calculated 95% CIs.

## Results

### Distribution of Google and Yelp Data

By using the Yelp API, we identified 8662 unique urgent care facilities that had 84,127 unique reviews. We collected 481,825 US-based reviews from Google Maps. Of these, 340,328 (70.63%) contained some text. The average star rating in Yelp reviews was significantly lower than the average star rating in Google Map reviews (*t*_565,950_=82.38; *P*<.001).

**Figure 1:**
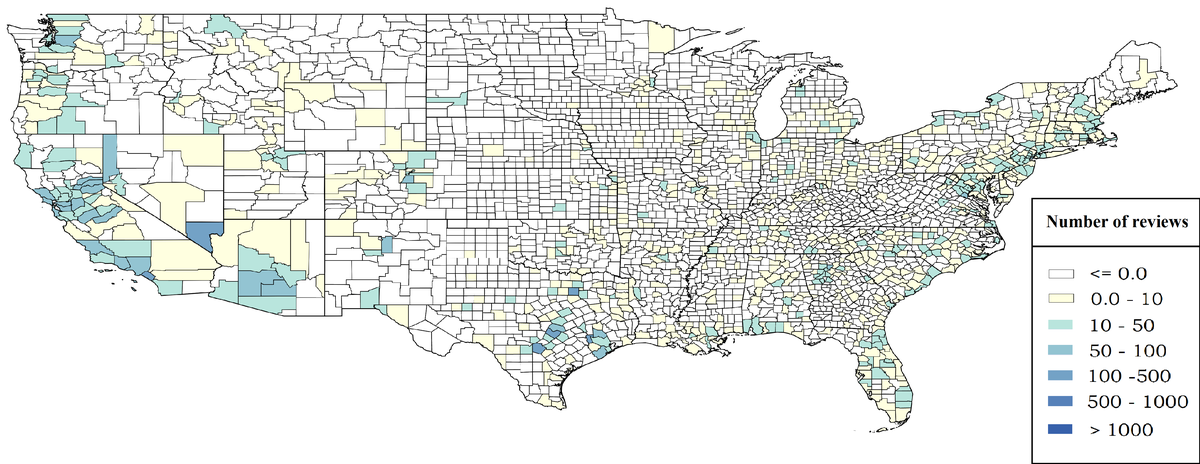
Total number of reviews in US counties (Yelp data).

**Figure 2:**
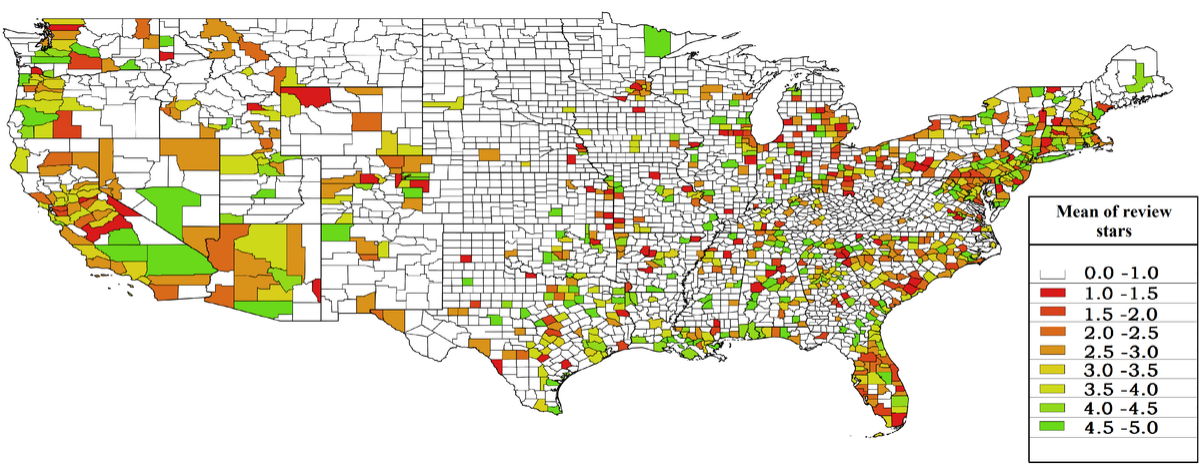
Mean review stars in US counties (Yelp data).

**Figure 3:**
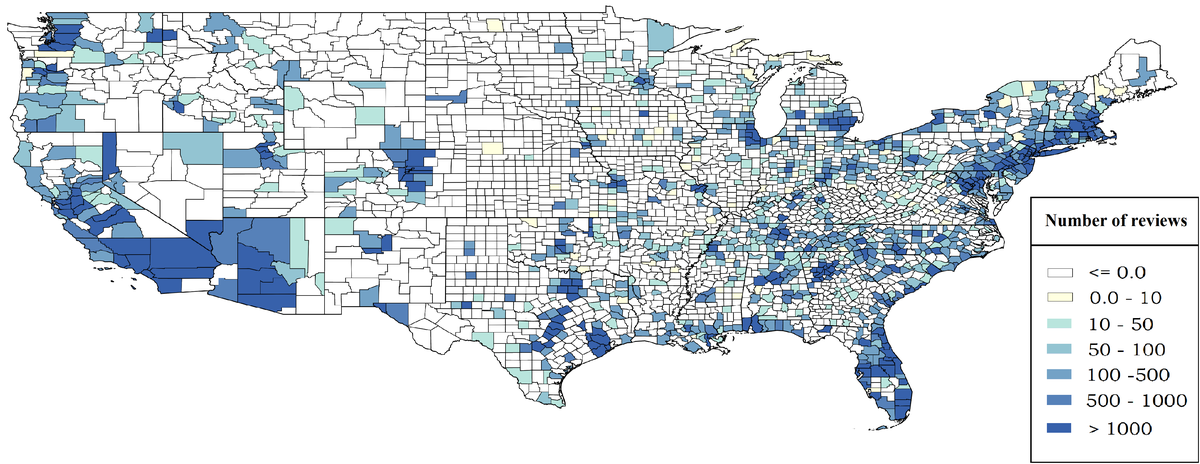
Total number of reviews in US counties (Google Maps data).

**Figure 4:**
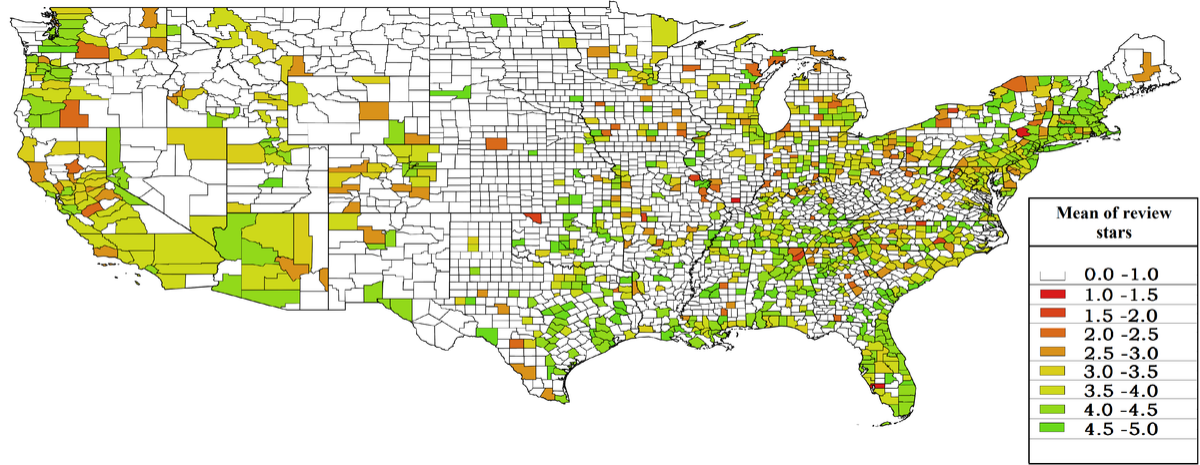
Mean review stars in US counties (Google Maps data).

Figures 1–4 display the distributions of the number of reviews and mean reviews stars from both Yelp and Google Maps on a 2-by-2 map. The distributions show an apparent difference between various geographical regions of the United States. The biased distribution is intriguing and can prompt many other research questions. Therefore, all state-by-state maps featuring the same information are being hosted on The George Washington University cloud, which can be made available to other researchers upon request.

**Figure 5:**
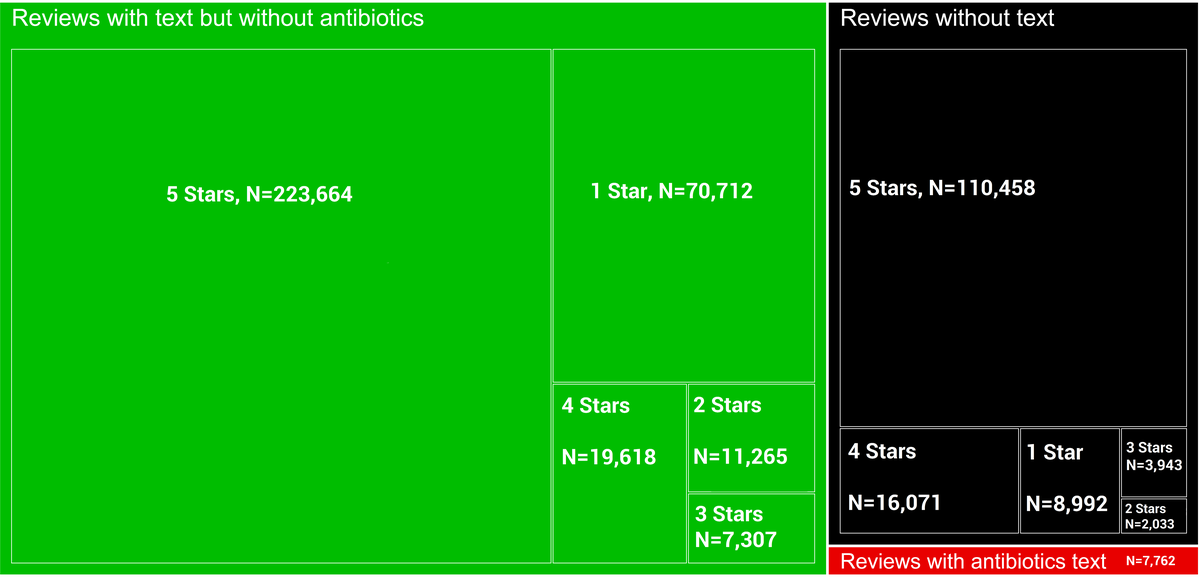
Distribution of Google reviews. Reviews with text but no antibiotic-related keywords are shown in green. Reviews with antibiotic-related keywords are shown in red. Reviews without text are shown in black.

We categorized the 481,825 reviews with text from Google Maps into the following three groups: (1) reviews without any text (n=141,497; star rating: mean 4.53); (2) reviews with text that did not mention antibiotic-related keywords (n=332,566; star rating: mean 3.94); and (3) reviews with text that mentioned antibiotic-related keywords (n=7762; star rating: mean 2.40; Figure 5). We found significant differences in the average star ratings across these three groups; a posthoc Tukey honestly significant difference test for multiple comparisons showed that reviews with antibiotic-related keywords had the lowest ratings compared to those in reviews without text (mean difference=−2.13; *P*<.001) and reviews with text but no keywords (mean difference=−1.54; *P*<.001). Reviews without text were significantly more positive than reviews with text but no keywords (mean difference=0.59; *P*<.001; all descriptive statistics are in Multimedia Appendix 4).

### Topic Modeling Results of Google Data

**Figure 6:**
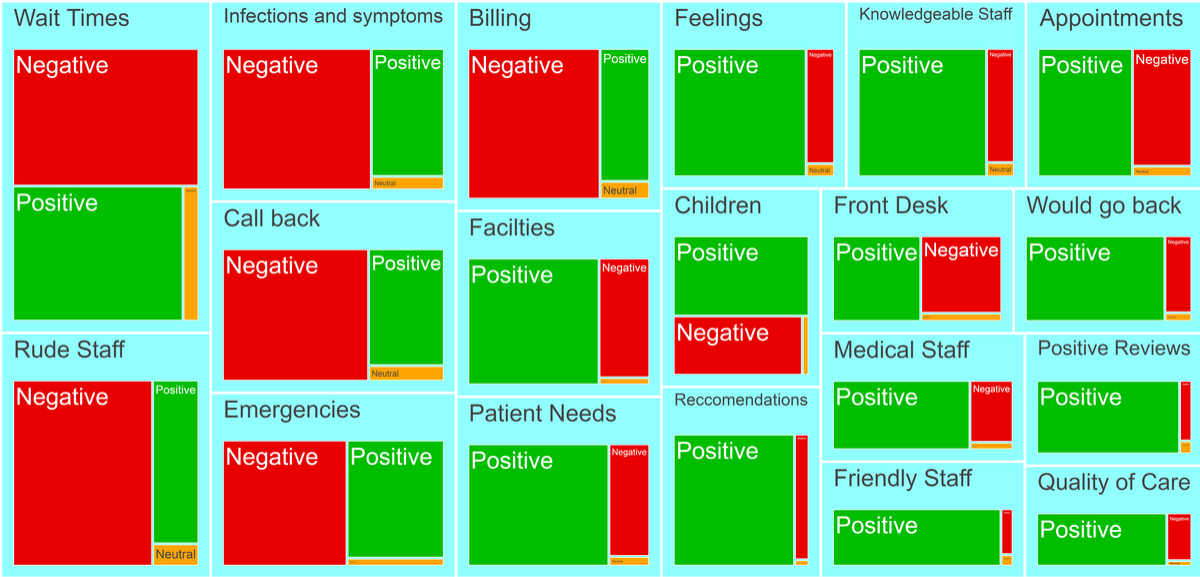
Topics generated by the latent Dirichlet allocation algorithm based on the text of all Google reviews in our data set. The size of each topic is proportional to the number of words that were assigned to each topic by the algorithm. Words are further segmented according to the sentiment of each review. Reviews with 4-5 stars are positive, reviews with 1-2 stars are negative, and reviews with 3 stars are neutral.

**Figure 7:**
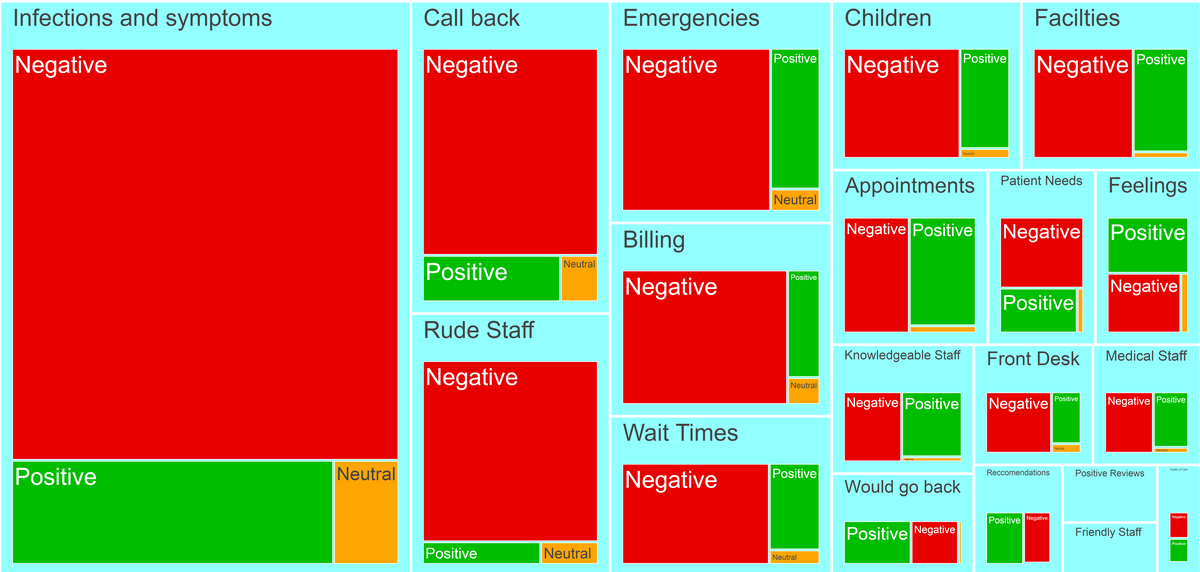
Subset of topic modeling results for reviews containing antibiotic-related keywords. The size of each topic is proportional to the number of words that were assigned to each topic by the algorithm. Words are further segmented according to the sentiment of each review. Reviews with 4-5 stars are positive, reviews with 1-2 stars are negative, and reviews with 3 stars are neutral.

The LDA topic modeling analysis (Figure 6; Multimedia Appendix 2) yielded 20 topics. The most negative topics pertained to rude staff, wait times, billing, callbacks, and other aspects of customer experience. Although the “infections and symptoms” topic was also predominantly negative, this topic did not make up a plurality of the reviews (481,760/6,795,468, 7.09%). With regard to topics with antibiotic-related keywords, Figure 7 shows that the most common topic pertained to infections and symptoms and was predominantly negative; however, several other topics pertaining to customer experience were also predominantly negative.

### Annotation Results

The annotators who labeled the seven inductive content categories achieved moderate reliability (Fleiss κ=0.42) with the first set of 100 reviews. All annotators agreed on sentiment. After disagreements were resolved, a second round of annotation for the next set of 100 reviews yielded substantial agreement (Fleiss κ=0.65), and disagreements were again resolved by consensus among reviewers. Table 1 summarizes the results of these annotations. Of a total of 200 reviews, we found that only 5 reviews (2.5%; 95% CI 0.3%-4.7%) exhibited the Yelp effect as the primary message category. By applying CIs to the full set of 8078 reviews containing antibiotic-related keywords, we expected that between 27 and 377 reviews would exhibit the Yelp effect as the primary message with 95% confidence. Thus, in our data set of 481,825 reviews, at most, 377 (0.08%) were expected to exhibit the Yelp effect.

**Table 1:** The seven primary message categories of the 200 messages.

Primary message	Reviews (N=200), n (%)
Yelp effect (the patient expected to receive antibiotics but did not receive an antibiotic)	5 (2.5)
Counter Yelp effect (the patient received antibiotics but did not want them)	1 (0.5)
Convenience, inconvenience, and wait times (positive and negative sentiment)	36 (18)
Staff competence or incompetence, courtesy and attitude, and satisfaction of care (positive and negative sentiment)	138 (69)
Cost and price of drugs per visit (including sticker shock; positive and negative sentiment)	13 (6.5)
Other complaints (positive and negative sentiment)	3 (1.5)
Other or none of the above	4 (2)

The annotators also reexamined all reviews to determine if they mentioned a Yelp effect in passing. We found that of a total of 200 reviews, 42 (21%) had some mention of reviewers not having received antibiotics when they were expected (Fleiss κ=0.75). Thus, with 95% confidence, between 1240 and 2152 of our 8078 reviews with antibiotic-related keywords exhibited the Yelp effect, even if it was only mentioned in passing. In our data set of 481,825 reviews, at most, 2152 (0.45%) were expected to exhibit the Yelp effect.

## Discussion

### Principal Findings

Our data suggest that the Yelp effect is quite rare. Out of a set of almost half a million reviews (N=481,824), fewer than 1 in 1250 (0.08%) seemed to contain the Yelp effect as the primary message (with 95% confidence). Furthermore, with 95% confidence, fewer than 1 in 225 (0.45%) reviews seemed to contain the Yelp effect as the primary or secondary message.

In contrast, we found that (138/200) 69% of the reviews in our annotated data set focused primarily on assessments of staff competence and the quality of personal interactions. This suggests that in terms of the extent that a Yelp effect exists, patients express this effect by questioning the expertise or personal qualities of urgent care staff. This may put urgent care providers in a bind; although they should not prescribe antibiotics inappropriately, a failure to explain to patients why the patients' preferred treatment is ineffective may lead to reviews that are designed to undermine care providers' credibility, expertise, and personal qualities. Thus, it is of paramount importance that both care providers and urgent care staff provide high-quality care and leave patients with a meaningful understanding of why they received the treatment that they did. For example, prior work has shown that patients' expectations for antibiotics are associated with categorical gist perceptions of the risks and benefits of antibiotics [26-28] and that patients more likely to be satisfied when they understand the gist of appropriate prescribing. This promotes the need to better communicate rationales for prescribing antibiotics in a manner that enhances patients' insights into why decisions are made and, by extension, their assessments of care providers' competence. Naturally, care providers' attitudes toward patient care are also important.

We aimed to answer the following question: is there a Yelp effect? The 2.28% (7762/340,328) of Google Maps reviews that mentioned antibiotics were indeed significantly more negative than those without antibiotic-related keywords (*P*<.001). Furthermore, our results show that reviews of urgent care centers on Yelp are significantly more negative compared to those on Google Maps (*P*<.001). Thus, we cannot rule out the existence of a Yelp effect on either Yelp or Google Maps. However, our results show that antibiotic prescription is merely one of the many potentially addressable issues in doctor-patient communication and may not be the primary source of negative web-based reviews. Indeed, patient satisfaction seems to have been most strongly linked to customer service issues (eg, wait times, rude staff, billing practices, etc). Thus, we must question whether claims regarding the impact of antibiotic prescriptions on negative reviews of urgent care centers are exaggerated. In recent years, some authors have suggested the presence of an effect that is similar to the Yelp effect in the context of opioid prescription [29,30]. However, similar to our findings, other studies have shown that these negative reviews are primarily comments on physicians’ attributes or administrative attributes [31].

### Limitations

The limitations of our work include our inability to hand-annotate all of the 481,825 reviews in our data set. Instead, we annotated 200 of the 7762 (2.58%) messages that were identified to have antibiotic-related keywords. This limitation was mitigated by the fact that these 200 messages were selected uniformly at random, meaning that they are likely to be representative of messages with antibiotic-related keywords. It is possible that our choice of keywords might have introduced selection bias; specifically, we assumed that patients who expected to (but did not) receive antibiotics would have said so in their reviews. Thus, we cannot rule out the possibility that patients were insincere when providing their reasons for negative reviews. However, our findings clearly indicate that patients were willing to express dissatisfaction with several other topics that do not directly pertain to antibiotics. Web-based reviews also often lack key patient information (eg, visit reason, medical history, and demographics). Finally, we do not claim that our results generalize beyond urgent care settings.

### Conclusion

Our analysis shows that the Yelp effect may not be a major driver of negative sentiments in web-based reviews. Rather than compromise medical and public health recommendations by acceding to the potentially faulty perceptions resulting from patients' desires, urgent care facilities should instead invest in efforts for improving patients' overall experience, such as reducing wait times, making billing practices transparent, and investing in training staff members to adhere to the best standards of customer service. Although these steps may not prevent all negative reviews, our analysis suggests that antibiotic prescribing need not be the focal point for patient satisfaction in urgent care settings.

## Appendix

Multimedia Appendix 1 Google Maps review collection algorithm.

Multimedia Appendix 2 Results and interpretations of 3 latent Dirichlet allocation models.

Multimedia Appendix 3 Antibiotic-related keywords.

Multimedia Appendix 4 Descriptive statistics.

## Abbreviations

API: application programming interface
LDA: latent Dirichlet allocation
