# Supplementary material for: Questioning the Yelp Effect: Mixed Methods Analysis of Web-Based Reviews of Urgent Cares
Source: J Med Internet Res. 2021 Oct 8;23(10):e29406. doi: 10.2196/29406 (PMC8538031; doi:10.2196/29406)
Supplement: Multimedia Appendix 1 [file jmir_v23i10e29406_app1.docx]

**Appendix 1 – Google Maps Reviews Collection Algorithm**

For each urgent care in our dataset, we retrieved the unique hash value assigned by Google Maps. For example, the School of Media and Public Affairs of GW has the following address on Google Maps:

<https://www.google.com/maps/place/George+Washington+University+School+of+Media+and+Public+Affairs/@38.8998658,-77.0482864,19z/data=!4m5!3m4!1s0x89b7b7b08af3296f:0x3e32dedd82d144b3!8m2!3d38.899823!4d-77.0461864>

In this case, the string “1s0x89b7b7b08af3296f:0x3e32dedd82d144b3” is the aforementioned hash value.

**Hiring Mturk workers with high performance to collect the URLs**

Since Google periodically updates these hash values, we hired human annotators to manually gather google URLs for our task. We conducted a pilot run in which we assessed Mturk workers’ performance. Specifically, we incorporated 100 URLs for which we already knew the correct response into each task. Workers who failed to submit the URLs in correct format were rejected. All Mturk workers are reimbursed for their work if they correctly submitted all URLs in the correct format. Ultimately, 20 workers were selected based on their performance on this pilot task. Each worker received $0.09 for each URL collected.

**Pseudocode for the crawling algorithms**

Each URL collected by an Mturk worker was used as input to a software program that we wrote to retrive the reviews from corresponding urgent cares. Here, we presented the pseudocode of our crawling algorithms. The code is composed of 4 main parts: crawl, parse, count and scroll. The core strategy is to expand the comment section thoroughly using a function that mimics human’s scroll and click action. After that, we parse and save all comment into an encrypted hard drive. All usernames are hashed during the crawling process to protect the privacy of all users. For each review, we obtained the following information: business index (to which urgent cares the review belongs), review text (if the user writes anything), stars (minimal star rating 1, maximum star rating is 5), state, and zip code. We also randomly generated a unique ID for each URL.

**def** crawl**(**self**,** url**):**

**try:**

assess the validity of the URL

**except** when the URL is not valid**:**

Print Exception

Ignore this URL

Continue on next URL

**try:**

assess the validity of the review section **:**

using SeleniumAPI the_driver**.**find_element_by_css_selector

**except** when the URL is not valid**:**

Print Exception

Ignore this URL

Continue on next URL

count_before_scroll ← Run Count function

Run Scroll Function

count_after_scroll ← Run Count function **(**#again after scroll#)

**while** count_after_scroll **>** count_before_scroll**:**

**(**# The scrolling will not stop until no more comment is available#)

count_before_scroll ← count_after_scroll

**(**#We updated the count_before_scroll#)

Run Scroll Function

**(**# Keep scrolling#)

count_after_scroll ← Run Count function

**(**#We updated the count_after_scroll#)

Click All more button using SeleniumAPI**:**execute_script

Run parse Function

**def** parse**(**self**):**

parse review_text

parse review_star

parse review_username

**def** count**(**self**):**

Count the current total of comments**.**

**return** num

**def** scroll**(**self**):**

Scroll the comment section Using SeleniumAPI**:** ActionChains

Pause Time **for** 15 seconds **(**# Timing Control**)**

**Code Availability**

Our code is available to researchers for legitimate academic research purposes only. Please contact the corresponding author if you would like to use our code.
