# Supplementary material for: Questioning the Yelp Effect: Mixed Methods Analysis of Web-Based Reviews of Urgent Cares
Source: J Med Internet Res. 2021 Oct 8;23(10):e29406. doi: 10.2196/29406 (PMC8538031; doi:10.2196/29406)
Supplement: Multimedia Appendix 3 [file jmir_v23i10e29406_app3.docx]

**Appendix 3 – Antibiotic Keywords**

antibiotic

anti-biotic

anti biotic

infect

strep

amox

augmentin

azithromycin

penicillin

sulfamethoxazole

trimethoprim

sulfa

bactrim

septra

ciprofloxacin

cipro

z-pak

z pak

z-pack

z pack

cefdinir

omnicef

clindamycin

clinda

cleocin

cephalexin

keflex
